# Evaluation of Monocarboxylate Transporter 4 in Inflammatory Bowel Disease and Its Potential Use as a Diagnostic Marker

**DOI:** 10.1155/2018/2649491

**Published:** 2018-05-08

**Authors:** Liying He, Hongli Wang, Yuhua Zhang, Lanlan Geng, Min Yang, Zhaohui Xu, Kejian Zou, Wanfu Xu, Sitang Gong

**Affiliations:** ^1^The First Affiliated Hospital of Jinan University, Jinan University, Guangzhou, China; ^2^Department of Gastroenterology, Guangzhou Women and Children's Medical Center, Guangzhou Medical University, Guangzhou, China; ^3^Department of Pediatrics, The Ninety-Five Hospital of PLA, Putian, China; ^4^Department of General Surgery, Hainan General Hospital, Haikou, Hainan, China; ^5^Guangzhou Institute of Pediatrics, Guangzhou Women and Children's Medical Center, Guangzhou Medical University, Guangzhou, China

## Abstract

**Background:**

Monocarboxylate transporter 4 (MCT4), encoded by SLC16A3 gene, is responsible for exporting lactic acid into the extracellular microenvironment, and an acidic microenvironment promotes cytokine production and remodels chronic inflammation, providing a link from glycolysis to inflammatory bowel disease (IBD).

**Objective:**

The aim of this study is to explore the value of MCT4 as a potential biomarker in IBD.

**Methods:**

The study group consisted of 39 cases with UC and 15 cases with CD. The centration of lactate level in serum was assessed by blood gas analysis, and MCT4 expression was analyzed by IHC.

**Results:**

Lactate level was increased in the forty-three of 54 patients (79.6%) with IBD by blood gas analysis compared with normal level (*P* < 0.001), in line with the result that showed increased MCT4 expression in inflamed colonic mucosa analyzed by immunohistochemistry. Most importantly, abundance of MCT4 expression was significantly associated with mucosal inflammation, which could be a clinical prognosis marker.

**Conclusion:**

The data suggested that increased lactate level in blood was possibly due to highly expressed MCT4 expression caused by inflammation in intestinal mucosal epithelial tissue, which could be a prognosis indicator of IBD in children.

## 1. Introduction

Inflammatory bowel disease (IBD), including Crohn's disease (CD) and ulcerative colitis (UC), is characterized as a group of immune-mediated disorders of the intestine [1]. Recently, a number of mouse models have been generated to focus on the interaction of metabolism and intestinal barrier function [2–5], for instance, AMPK improves gut epithelial differentiation and barrier function via regulating caudal-type homeobox transcription factor 2 (CDX2) expression, and fatty acid synthase (FASN) modulates intestinal barrier function through palmitoylation of mucin 2, while sirt5 desuccinylates and activates pyruvate kinase M2 (PKM2) to block macrophage IL-1beta production and to prevent DSS-induced colitis in mice. These finding indicated the critical role of metabolism in the development of IBD.

In particular, IL-10^−/−^ mice were characterized by higher levels of lactate, pyruvate, and citrate and lower glucose, which suggested increased fatty acid oxidation and glycolysis [6], suggesting that IBD is close with metabolic disorders. Application of the well-established metabonomic approach could help to define early biomarkers of pathogenesis to be used for disease surveillance. Metabolomics indeed provides an efficient way to diagnose toxicological and pathophysiological states, assess metabolic response to environmental factors, and characterize metabolic phenotypes of mammals including host and gut microbiome metabolic interactions and the metabolic effects induced by nutritional interventions [7, 8].

Aerobic glycolysis, a phenomenon described by Otto Warburg almost 90 years ago, is a hallmark of cancer [9] and has been pointed out as a promising target for anticancer therapy [10]. Interestingly, Qu et al.'s study showed that inflammation, characterized by IBD, upregulated key glycolytic enzyme expression via activation of STAT3/c-myc signaling pathway within dextran sulfate sodium- (DSS-) induced mouse colitis model [11], indicating cell metabolism has been reprogramed during the process from IBD to CRC. Moreover, higher level of lactate suggested increased glycolysis [6]. However, little information is known about the role of MCT4, which is responsible for lactate transport, in IBD.

MCT4, a low-affinity and high-capacity lactate transporter that is present in cells exhibiting elevated glycolytic activity, is involved in lactate release from glycolytic cells. It plays an essential role by contributing both to the acid-resistant phenotypes of cancer cells, by mediating lactate and proton efflux to the extracellular milieu [12].

The human monocarboxylate transporter 4 (MCT4) is strongly expressed in glycolytic tissue, such as skeletal muscle fibers, astrocytes, leukocytes, chondrocytes, and some mammalian cell lineages [13, 14]. They promote the efflux of lactic acid, constituting important players in the maintenance of tumor intracellular pH, as well as in the maintenance of the high rates of glycolysis [15–18]. What is more? Thibault et al. have demonstrated that monocarboxylate transporter 1 (MCT1) is downregulated in inflamed colonic mucosa of IBD patients and rats [19–21]. Despite the strong research concerning the function of MCT4 in tumor, data are lacking in inflammatory bowel disease, especially in children. In this study, we aimed to seek evidence to elucidate the possible relationship between MCT4 expression and IBD development/prognosis.

## 2. Materials and Methods

### 2.1. Patients and Biopsies

Based on the declaration of Helsinki as reflected in a prior approval by the institution's human research committee, this study was conducted in a cohort of child patients with inflammatory bowel disease (IBD) in Guangzhou Women and Children's Medical Center from 2004 to 2015 approved by the Medical Ethical Review Board, named Scientific Research Committee of Guangzhou Women and Children's Medical Center at March 10, 2017.

A total of 54 patients with IBD were included in this study; the detailed information was supplied in Supplementary Materials S1. The intestinal tissue was drawn from each patient by electronic colonoscopy after we got the informed consent from the patients diagnosed with IBD. Written informed consent was given by the caregiver of the child for his clinical records used, which are not publicly available since the database is currently not anonymous and contains all patient's name; however, it could be available upon request.

### 2.2. Blood Gas Analyses

Blood samples were drawn from patients for lactate measurement (GEM Premier 3000 analyzer) in clinical laboratory.

### 2.3. Immunohistochemistry

Colonic tissue from patients was routinely formalin-fixed and paraffin-embedded and then cut into slices of 4 mm for immunohistochemical staining. To test the MCT4 expression in intestinal epithelium of IBD patients, the sections were processed with the following steps. Slices were placed in 55°C oven for 2 hours and then deparaffinized in xylene and in gradient alcohol solutions. 1 × citrate buffer (heated to boiling) was used to antigen retrieval. When cooled to room temperature, the hydrogen peroxide (3%) was used to inactivate endogenous peroxide activity and blocked in goat serum at room temperature for 40 min, followed by incubation with anti-MCT4 (1 : 500) antibody overnight at 4°C. After colorized with diaminobenzidine (DAB Kit; ZSGB-BIO Co.) and counterstained with hematoxylin, sections were dehydrated in gradient alcohol solutions and in xylene. Sections mounting of the coverslips were photographed by microscopy.

### 2.4. Statistical Analysis

All statistical analyses were performed using SPSS 22.0 (SPSS Inc., Chicago, IL). Data were expressed as the mean with standard deviation (SD). One-sample *t*-test was used to analyze the difference of lactate level. All statistical analyses utilized a 0.05 level of significance.

## 3. Results

### 3.1. Patient's Characteristics

As shown in Table 1, the patients in this study consisted of 39 (72.2%) boys and 15 (27.8%) girls with an age at diagnosis of 1 to 16 years (median, 8 years) in Guangzhou Women and Children's Medical Center; with regard to IBD grade (consisted of 40 cases CD (74.1%) and 14 cases UC (25.9%)), patients were classified as active and remission stage, 68.5% and 31.5%, respectively. In addition, according to the degree of mucosal inflammation, 24 cases (44.4%) are moderate, and 17 cases (31.5%) and 13 cases (24.1%) were mild and severe, respectively.

### 3.2. Lactate Level Is Increased during Intestinal Inflammation

**Figure 1:**
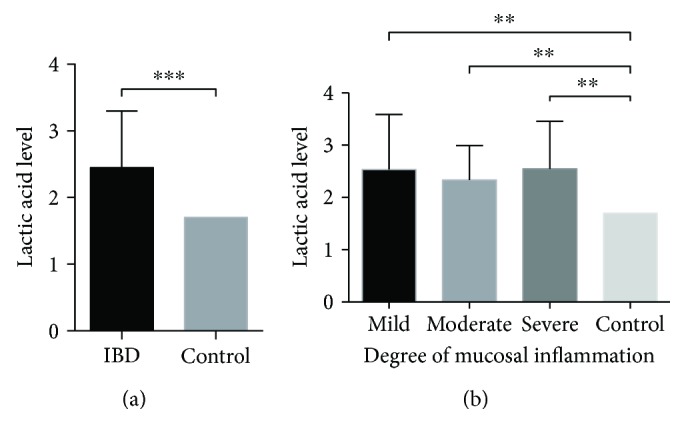
Lactic acid level was measured in patients with IBD. (a, b). Lactic acid level was measured by blood gas analysis, and statistical difference was analyzed by one-sample *t*-test. ^∗∗^ *p* < 0.01 and ^∗∗∗^ *p* < 0.001.

Data analysis of lactate was compared with control subjects and divided into two sections; thirty-three of the 40 (82.5%) patients with CD and ten of the 14 (71.4%) patients with UC had significant increased lactate phenomenon by blood gas analysis. What is more? The lactate level in IBD patients, including UC and CD, has significant difference compared to the normal level *in vitro* (Figure 1(a), 2.451 ± 0.85 versus 1.7; *p* < 0.001). In addition, based on the degree of mucosal inflammation, we found that forty-three of 54 patients (79.6%) with lactate increased, but no difference among IBD grades (Figure 1(b)), which is attributed to various cells, including inflammatory cell, could produce lactate. In line with a study reported by Wang et al., HG and IL-1b decrease MCT4 and its location on plasma membrane as well as increase lactic acid accumulation and apoptosis in HUVECs [22], suggesting the critical role of inflammation in regulation of MCT4 expression. However, the expression of MCT4 in inflammatory bowel disease remains poorly understood.

### 3.3. MCT4 Expression Is Increased in Intestinal Mucosal Epithelial Cell in IBD

**Figure 2:**
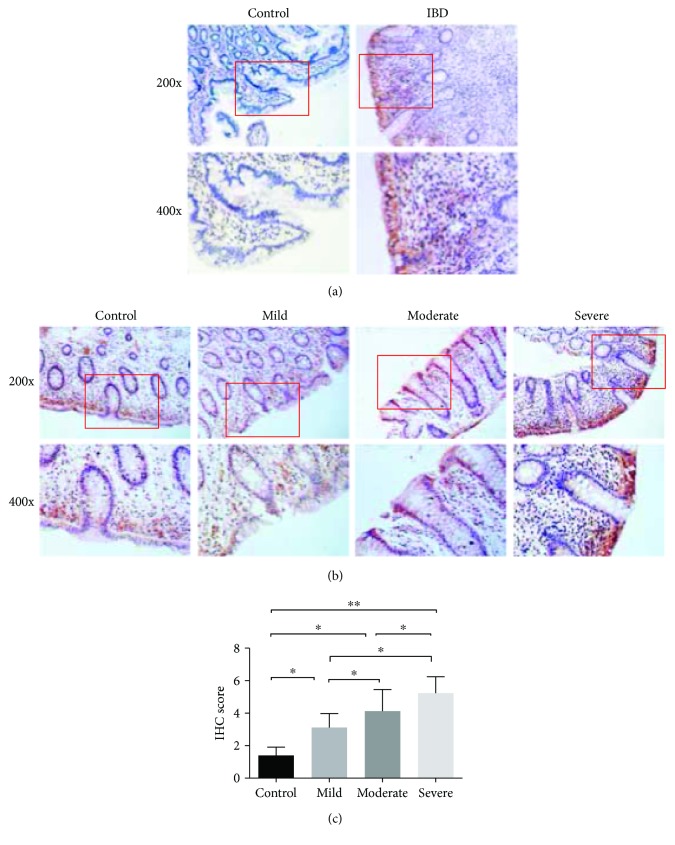
MCT4 expression is increased in IBD tissue. IHC staining was performed with anti-MCT4 antibody in intestinal tissue as indicated group (a, b). ^∗^ *p* < 0.05 and ^∗∗^ *p* < 0.01.

The above results showed that lactate is increased in patients with IBD, which is transported across cell membranes by MCT4. We next sought to verify whether the upregulation in MCT4 expression was proportional to the degree of mucosal inflammation. We first confirmed that MCT4 is primary located in the intestinal mucosal epithelial tissue by IHC analysis (Figure 2(a)), which is attributed to the function of MCT4. In addition, endoscopic estimation of the inflammatory status of colonic biopsy specimens by analysis indicated that MCT4 expression is a closely mucosal inflammation (Figure 2(b)). What is more, MCT4 expression levels were significantly higher in inflamed biopsy specimens than in noninflamed or control biopsy specimens, implying that the degree of MCT4 expression is positively close with mucosal inflammation; interestingly, MCT4 expression was correlated with IBD grade of severity (Figure 2(c)). All these findings suggested that MCT4 could be a prognostic indicator of IBD.

## 4. Discussion

A number of original studies that investigated the clinical and prognostic significance of MCT4 have been conducted in various types of cancers, such as lung cancer, prostate cancer, and colorectal cancer [23–27], suggesting a functional role of the MCT4 in tumor progression. However, the expression of MCT4 and its clinical value in IBD are still poorly understood. This is the first study, to our knowledge, investigating the expression and clinical prognostic significance of MCT4 in IBD. The results showed that MCT4 expression is mainly in intestinal epithelial cells. Importantly, the immunohistochemical results showed that increased expression of MCT4 in patients with IBD, which is significantly associated with IBD progress, finally results in promoting of the lactate production. It could be as a prognostic indicator of IBD.

MCT4 has been observed in lactate-producing tissues, specifically in skeletal muscle and astrocytes, and displays a lower affinity for substrates such as L-lactic acid and pyruvic acid. Recently, MCT4 has been shown to localize to the membrane of glial cells in the cerebellum [14] and the paraventricular nucleus, specifically in astrocytes and ciliated ependymal cells, playing a significant nonmetabolic role in the neuroprotective mechanism of ischemic preconditioning (IPC) in the gerbil with transient cerebral ischemia [28]. In addition, MCT4 expression was very weak in lymphocytes and was limited to the plasma membrane, which is similar to those of granulocytes with MCT2>MCT1>>>MCT4 [29]. The function of MCT4 in various diseases remained to be fully elucidated.

Surprisingly, we found that MCT1 was also highly expressed in intestinal epithelial cells of IBD patients analyzed by IHC (Supplementary Figure 1), which is not in line with Thibault et al.'s study that MCT1 is downregulated in inflamed colonic mucosa of IBD patients and rats by IF [20]. We also found in Thibault et al.'s study that in DSS-induced colitis model, Western blotting was performed to detect MCT1 expression, which not only includes intestinal epithelial cells but also inflammatory cells and fibrocytes. In addition, the limitation of tissue captured by endoscopy failed to isolate intestinal epithelial cells. Based on these questions, further work is required to address.

Our previous study demonstrated that miR-1 suppresses smad3-mediated MCT4 expression in tumor glycolysis in colorectal cancer [30]. Also, recent studies showed that MCT4, directly mediated by HIF-1*α*, maintains a high level of glycolysis, and the enhanced glycolysis promotes proinflammatory properties [31]. Lactate, the end-product of glycolysis, is released into the extracellular environment. The acidic microenvironment promotes the production of proinflammatory cytokines, which bridges the gap between chronic inflammation and cancer development [32]. These findings suggested that the accumulation of lactate in solid tumors is a pivotal and early event in the development of malignancies. In addition to risk factors for colorectal cancer in IBD, which correlates with the duration of the disease, extent of the disease, the association with primary sclerosing cholangitis, family history, and early age at onset [33], interestingly, we found that MCT4 is significantly increased in patients with IBD compared with healthy control, which may cause an increased risk of developing intestinal cancers. Further work is required to discuss whether the MCT4 inhibitor could stop the development of IBD, and it also will provide novel avenues for the management of IBD.

Lactate, a production of cancer-associated metabolic reprogramming, can be triggered during the process of chronic inflammation [11, 34–36], termed as colitis-associated cancer in clinical diagnosis. This metabolic characteristic differing from normal cells might be a novel diagnosis biomarker of chronic colitis-associated cancer or even as novel potential therapeutic targets for IBD. Taken together, our results showed that lactate level is increased in patient with IBD, which may be attributed to increased MCT4 caused by chronic inflammation. Despite the functional role of MCT4 in IBD remains poorly understood, its expression is associated with clinical prognosis.

## Figures and Tables

**Table 1:** The characteristic of the inflammatory bowel disease patients.

Variables	Number of patients (%)	Lactic acid level^∗^ (%)	
		Increased	Normal
Total	54	43 (79.6)	11 (20.4)
Age			
<10 years	32 (59.3)	26 (48.2)	6 (11.1)
≥10 years	22 (40.7)	17 (31.4)	5 (9.3)
Gender			
Male	39 (72.2)	30 (55.5)	9 (16.7)
Female	15 (27.8)	13 (24.1)	2 (3.7)
Type of IBD			
Crohn's disease	40 (74.1)	33 (61.1)	7 (13.0)
Ulcerative colitis	14 (25.9)	10 (18.5)	4 (7.4)
Stage			
Active stage	37 (68.5)	30 (55.5)	7 (13.0)
Remission stage	17 (31.5)	13 (24.1)	4 (7.4)
Degree of mucosal inflammation			
Mild	17 (31.5)	13 (24.1)	4 (7.4)
Moderate	24 (44.4)	19 (35.1)	5 (9.3)
Severe	13 (24.1)	11 (20.4)	2 (3.7)
Therapy			
Hormone	26 (48.2)	23 (42.6)	3 (5.6)
Immunosuppressant	8 (14.8)	6 (11.1)	2 (3.7)
Hormone + immunosuppressant	20 (37.0)	14 (25.9)	6 (11.1)

^∗^Normal level is 0.9–1.7.

## Supplementary Materials

Supplementary 1 Supplementary materials S1: the detailed information of 54 patients were listed.

Supplementary 2 Supplementary Figure 1: MCT1 expressions were elevated in intestinal mucosal detected by IHC.
