# Supplementary figures and images for: Evaluation of Monocarboxylate Transporter 4 in Inflammatory Bowel Disease and Its Potential Use as a Diagnostic Marker
Source: Dis Markers. 2018 May 8;2018:2649491. doi: 10.1155/2018/2649491 (PMC5964618; doi:10.1155/2018/2649491)

## MCT1

healthy control

IBD group

Case1

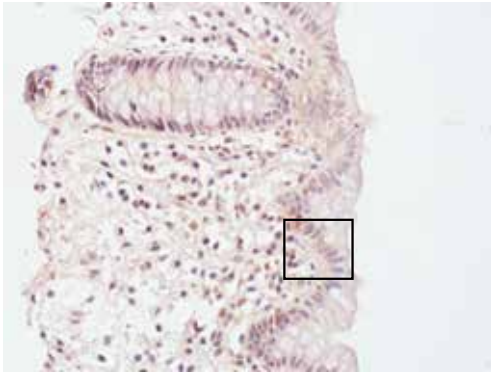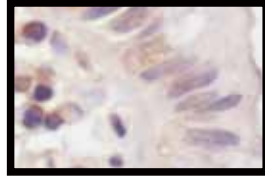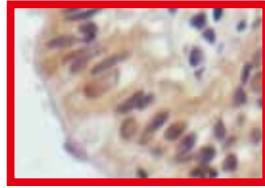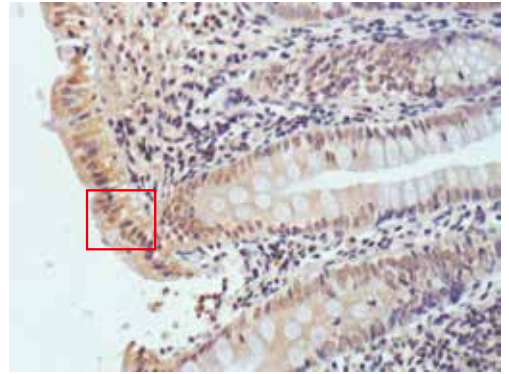

Case2

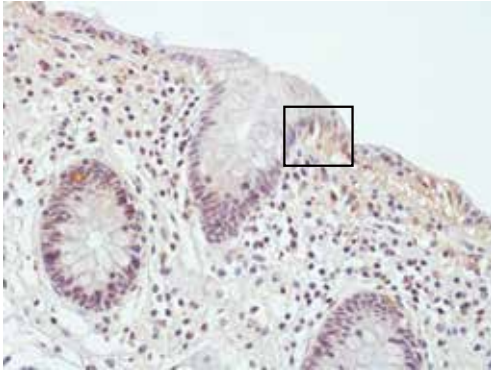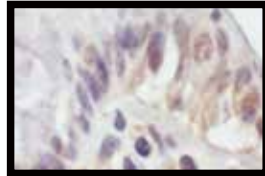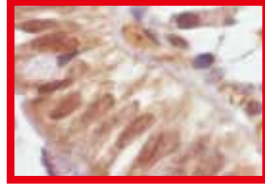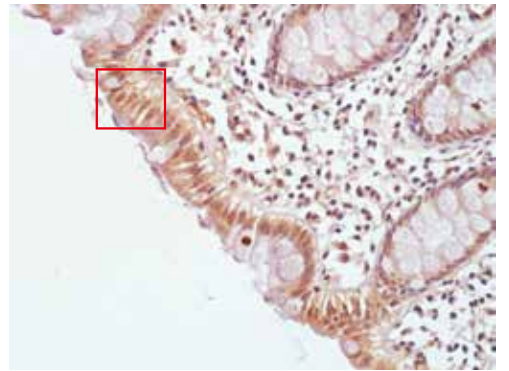

Supplement: Supplementary 2 — Supplementary Figure 1: MCT1 expressions were elevated in intestinal mucosal detected by IHC. [file 2649491.f2.pdf]
